# Multilevel Proteomics Reveals Epigenetic Signatures in BCG-Mediated Macrophage Activation

**DOI:** 10.1016/j.mcpro.2024.100851

**Published:** 2024-10-02

**Authors:** Zoe Schaefer, John Iradukunda, Evelyn N. Lumngwena, Kari B. Basso, Jonathan M. Blackburn, Ivana K. Parker

**Affiliations:** 1 Department of Biomedical Engineering, University of Florida, Gainesville, Florida, USA; 2 Division of Chemical & Systems Biology, University of Cape Town, Cape Town, South Africa; 3 School of Clinical Medicine, University of The Witwatersrand, Johannesburg, South Africa; 4 Center for the Study of Emerging and Re-emerging Infections (CREMER), Institute for Medical Research and Medicinal Plant Studies (IMPM), Ministry of Scientific Research and Innovation, Yaounde, Cameroon; 5 Mass Spectrometry Research and Education Center, Department of Chemistry, University of Florida, Gainesville, Florida, USA

**Keywords:** trained immunity, THP1, macrophage, BCG, mycobacteria, multi-omics, proteomics, mass spectrometry, epigenetics

## Abstract

The *bacillus* Calmette-Guérin BCG vaccine (*Mycobacterium bovis*) is primarily used to prevent tuberculosis (TB) infections but has wide-ranging immunogenic effects. One of its most notable properties is its ability to induce trained immunity, a memory-like response in innate immune cells such as macrophages. Through targeted analyses of well-established histone marks, prior research has shown that these changes are generated through epigenetic modification. Mass spectrometry-based proteomic approaches provide a way to globally profile various aspects of the proteome, providing data to further identify unexplored mechanisms of BCG-mediated immunomodulation. Here we use multi-level proteomics (total, histone, and phospho to identify networks and potential mechanisms that mediate BCG-induced immunomodulation in macrophages. Histone-focused proteomics and total proteomics were performed at the University of Cape Town (data available via ProteomeXchange with identifier PXD051187), while phosphoproteomics data was retrieved from the ProteomeXchange Repository (identifier PXD013171). We identify several epigenetic mechanisms that may drive BCG-induced training phenotypes. Evidence across the proteomics and histone-focused proteomics data set pair 6 epigenetic effectors (NuA4, NuRD, NSL, Sin3A, SIRT2, SIRT6) and their substrates.

## Highlights

• Multilevel proteomics analysis reveals diverse whole-cell changes.
• BCG stimulation is associated with altered levels of proteins involved in diseases.
• A network of epigenetic effectors drives BCG-induced macrophage activation.

## Graphical Abstract

**Figure:**
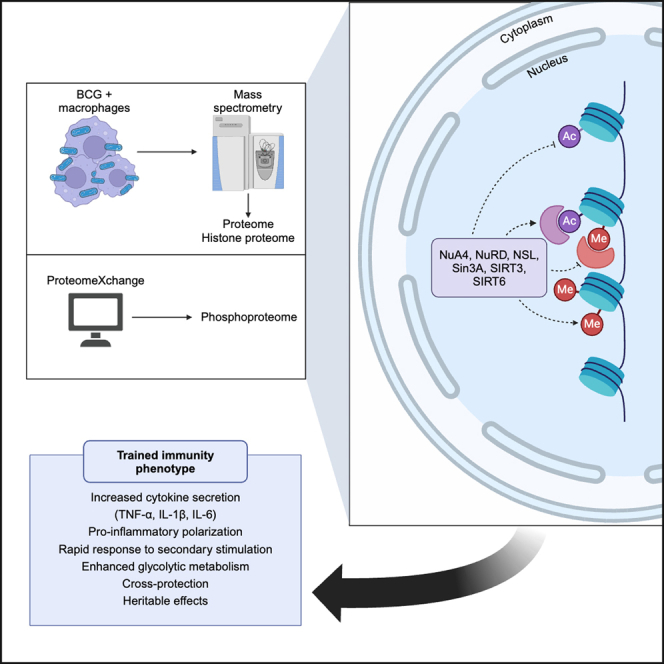

## In Brief

This study investigated BCG-induced macrophage activation with multiple levels of proteomic analysis, including total proteome, phosphoproteome, and proteome of isolated histones. Using an integrative systems biology approach, we identified a network of epigenetic effectors and their substrates that changed in prevalence after BCG infection. These results provide insight into the epigenetic mechanisms that drive trained immunity and identify avenues for further research into leveraging it in a clinical setting.

The *bacillus* Calmette-Guérin (BCG) is a live, attenuated *Mycobacterium bovis* administered to prevent tuberculosis (TB) transmission in endemic settings. Globally, BCG has shown varying efficacy as a TB vaccine, with rates ranging from 0 to 80% (1). Recent studies have found significant protection against TB in cohorts of children aged 0 to 5 (2), but such benefits have not been observed in adolescents and adults (3). Beyond its role in TB prevention, BCG has proven to have other advantages. Notably, vaccinated infants have shown a reduction in all-cause neonatal mortality (4, 5). Furthermore, BCG administration is associated with increased resistance to viral infections (6) and efficacy in treating some forms of bladder cancer (7). Despite these beneficial aspects, BCG can also have deleterious outcomes. Studies indicate an elevated risk of mother-to-child transmission of HIV among BCG-vaccinated infants (8). Additionally, BCG vaccination in HIV-positive children can lead to immune reconstitution inflammatory syndrome (IRIS) (9).

Studies to investigate BCG’s immunomodulatory properties have led to the discovery of its ability to induce trained immunity (10, 11) and create a long-lasting, memory-like response in innate immune cells not traditionally associated with immunological memory. Subsequently, trained cells display increased activation when exposed to a stimulus unrelated to the original pathogen. Of these, macrophages are an immune cell type trained by BCG; several studies examine and characterize BCG-training modalities within macrophages (10). Trained phenotypes can vary based on specific parameters such as cell type, length of exposure, initial stimulus, and secondary stimulation. In BCG-vaccinated adults, macrophages trained by BCG respond more intensely to *Candida albicans* with higher secretion of TNF-α and IL-6 (12). *In vitro* and *in vivo* studies show that trained cells also exhibit a shift towards glycolytic metabolism (13). Altered pathways can include polarization to a pro- or anti-inflammatory phenotype, increased cholesterol synthesis, and endotoxin tolerance (13, 14); however, the precise changes in pathway regulation vary based on the training stimulus.

Targeted experiments have shown that these changes in cellular function are regulated by epigenetic modifications that occur at the histone level (15). For instance, the pro-inflammatory cytokine response associated with trained immunity is primarily regulated by genome-wide enrichment in histone 3 lysine 27 acetylation (H3K27ac) and concentration of histone 3 lysine 4 trimethylation (H3K4me3) at promoters for *TNF* and *IL-6* (16, 17). Additionally, trained but resting macrophages accumulate H3K4 monomethylation at promoters, indicating “poised” genes (14). Connecting histone “codes” to training states provide mechanisms to explain undesired consequences of BCG (10), and more comprehensive studies can reveal targets for improved therapeutics that reduce off-target effects.

Within the host cell, *Mycobacteria* alter signaling pathways to promote their survival and pathogenesis (18). Given that signaling cascades involve the expression and modification of numerous proteins, proteomics approaches provide a unique advantage for probing cellular changes. Used in concert, shotgun proteomics allows for global profiling of peptides, while targeted proteomics can evaluate post-translational modifications at the histone level (19). Previous studies examined epigenetic regulation of trained immunity by examining well-defined histone marks (16, 17). As cellular function is hypothesized to be mediated by a histone code, global profiling of histone post-translational modifications that mediate gene expression is important to characterize. Furthermore, the cellular machinery that regulates histone modifiers can be phosphorylated to promote or suppress activity. These highlight the ability of phosphoproteome data to provide another level of mechanistic probing into factors that direct BCG-trained immunity. Altogether, this multi-level proteomics approach is a powerful tool to fully elucidate mechanisms of trained immunity.

Here, we present a multilevel view of the BCG-infected macrophage proteome, epiproteome, and phosphoproteome. Using label-free proteomic studies in human cells, we identify key common pathways that influence trained immunity and BCG pathology. We additionally support these findings through analysis of publicly available data of tandem mass tag (TMT)-based phosphoproteomics. Given that TB and HIV are co-endemic in many regions of the globe, it is essential that the positive and negative effects of BCG-stimulation are decoupled. Proteomics-based systems-level analyses of BCG-macrophage interactions provide data context to unveil critical mechanisms mediating trained immunity.

## Experimental Procedures

### Experimental Design and Statistical Rationale

The purpose of this study was to determine immunogenic targets of interest in BCG-macrophage interactions and characterize epigenetic modifiers that may direct cellular outcomes. Two datasets were collected to study the total and histone proteome, while a third dataset was retrieved from the PRIDE database that represented the phosphoproteome of macrophages stimulated with BCG. All datasets examined macrophage activation after BCG stimulation using a THP-1 cell line. For the total proteomic and histone-focused proteomic sets, Fisher’s exact test (pairwise ratio-based) was used to calculate *p*-values with low-intensity resampling value imputation included. Adjusted *p*-values were calculated using the Benjamini-Hochberg method. For the phosphoproteomics data, as described by Choudhary *et al*., a two-tailed Student’s *t* test was used to calculate *p*-values for normally distributed data. Pathway enrichment (in R and Metascape) also uses the Benjamini-Hochberg False Discovery Rate (FDR) to determine statistical significance. The total and histone-focused proteomics experiments were performed in groups (infected and control) of 5 biological replicates. One replicate was eliminated from each group in the total proteomics experiment due to unusually low output, resulting in a group size of four. The histone-focused groups each had five samples.

### BCG Culture

All work was aseptically performed under BSL-2 containment. Bacteria were cultured in a shaking incubator (120 RPM) at 37 °C. Culture media was Middlebrook 7H9 broth (Becton Dickinson) with 0.2% glycerol, 0.05% Tween 80, and 10% OADC (Becton Dickinson) supplement.

The *M. bovis* BCG culture (Pasteur) was grown from frozen stocks (stored at −80 °C). Thawed BCG stocks were inoculated (0.1% v/v) into 5 ml pre-warmed 7H9 culture media in a T-25 flask and grown to mid-log phase (OD_600_ = 0.8). Broth culture was streaked for single colonies onto Middlebrook 7H10 agar and incubated at 37 °C for 4 weeks. Single colonies were harvested and inoculated into 25 ml of pre-warmed 7H9 culture media in a 125 ml conical flask and incubated to mid-log phase (OD_600_ = 0.8). Thereafter, 0.1% of these cultures were inoculated into 100 ml of warmed media in 500 ml conical flasks and left to grow to the desired OD_600_ value of 0.8. For infection, bacterial clumps were disrupted using a water bath sonicator for 10 min before being passed 10x through a 22-gauge needle, followed by gentle centrifugation (100g for 10 s) to sediment clumps. The supernatant was then transferred to a separate tube and absorbance was read using a spectrophotometer.

### THP-1 Culture

Human THP-1 monocytes (ATCC) were cultured in a humidified atmosphere with 5% CO_2_ at 37 °C, in RPMI-1640 (supplemented with 0.05 mM 2-mercaptoethanol, 2 mM L-glutamine, 10% heat-inactivated fetal bovine serum, 50 IU/ml penicillin, and 50 μg/ml streptomycin). To differentiate monocytes into macrophages, cells were added to T300 flasks at a density of 1 million cells/ml and incubated with 100 nM phorbol 12-myristate 13-acetate (PMA) for 24 h, followed by a 24-h rest in PMA-free media.

### Macrophage BCG Infection

Bacterial cells, at mid-log phase (OD_600_ = 0.8), were washed twice with PBS, and resuspended into antibiotic-free RPMI culture media, yielding an infection buffer. Macrophages were washed twice with PBS and incubated in an infection buffer at an MOI of 4:1 for 24 h. Cells were rinsed twice with PBS and lysed on ice for subsequent proteomic analysis. Lysis buffer consisted of 1% sodium dodecyl sulfate, 1.5% sodium deoxycholate, 50 mM Tris, Benzonase endonuclease, and additional protease and phosphatase inhibitors.

### Total Proteomics

For total proteomics, cells were lysed, proteins precipitated, digested, and desalted after collection as previously described (20). Briefly, proteins were quantified with a BCA assay. Proteins were denatured with 1 mM dithiothreitol (DTT) for 1 h at room temperature with gentle agitation and alkylated with 5.5 mM iodoacetamide (IAA) for 1 h in the dark. Proteins were pre-digested with Lys-C endopeptidase (Wako) for 3 h at 30 °C before being diluted four times with HPLC-grade water to a final concentration of ∼50 mM ammonium bicarbonate (ABC). The diluted sample was then digested overnight with trypsin (1:100 ratio) at 37 °C. The digestion was quenched with 1% trifluoroacetic acid (TFA) (Sigma Aldrich).

### Histone Proteomics

Histones were isolated as described previously (21, 22). Briefly, cells were precipitated by adding acetone/methanol (8:1) and incubating overnight at −20 degrees. Nuclei were isolated from macrophages and histones were acid extracted (0.2 M H_2_SO_4_), precipitated (TCA to a final concentration of 33%), and washed with ice-cold 100% acetone. Proteins were quantified with a BCA assay and propionylated for two rounds to preserve lysine post-translational modifications during tryptic digest. Peptides were resuspended in 50 mM ABC to achieve a concentration of 1 μg/μl. Trypsin was added in a 1:10 ratio and incubated at 37^o^ C for 6 to 8 h. Digestion was stopped via freezing at −80 °C. Histone peptides were again propionylated to improve retention of short peptides at the N-termini.

### Desalting

For both total and histone proteomics, digested peptides were desalted to remove salts accumulated during in-solution digestion. Firstly, C18 disks (Millipore) were activated with 2 × 100 μl Solvent B (Methanol), centrifuging at 1000 g with 30 s intervals, making sure that the membrane remained wet, then equilibrated twice with Solvent A (2% ACN and 0.1% FA) prior to addition of sample to the disk. Sample-bound disks were then washed twice with Solvent A, followed by slow elution (at 500g) of peptides with Solvent C (60% ACN and 0.1% FA) into a glass insert, after which peptides were vacuum dried at room temperature in a SpeedyVac (Savant). Dried peptides were then resuspended in Solvent A to a final concentration of 200 ng/μl before LC-MS/MS analysis.

### LC-MS/MS Measurement

Peptides were loaded onto a Dionex RS3500 (Thermo Fisher Scientific) coupled to a Q Exactive mass spectrometer (Thermo Fisher Scientific). Peptides were loaded onto a 75 μm (ID), 15 cm column packed in-house with reversed-phase Phenomenex core-shell 1.7 μm resin (Phenomenex). Peptides were then eluted using a 50-min linear gradient of solvent B (100% ACN in 0.1% formic acid) from 2%–6% for 2 min, 6%–23% for 48 min using curve 8 at 400 nl/min followed by a washout at 80% for 10 min. Full scans were recorded between 300 to 1750 Thompson at a resolution of 70,000 with an AGC target of 3e^6^. For proteome measurements, the 10 most intense ions from each full scan were selected for fragmentation (MS/MS) by higher-energy collisional dissociation (HCD) using an NCE of 28 and an AGC target of 1e^5^ in 80 ms at a resolution of 17,500. All raw and spectral search output data was deposited to ProteomeXchange (23) and is freely available under the identifier PXD051187.

### Protein Identification and Data Handling

#### Histone-Focused Proteomics

Mass-spectrometry data was processed using EpiProfile version 2.1 (24) in MATLAB R2022a. EpiProfile is optimized for data-independent acquisition through the use of precursor and fragment-extracted ion chromatography to accurately determine the chromatographic profile and to discriminate isobaric forms of peptides. EpiProfile was set to detect human species and assessed with label-free quantification with a peptide mass tolerance of 10 ppm, Briefly, unmodified fragments of each replicate are isolated and used to exclude fragment outliers (peptides must be within 2 min of the reference retention time). Modifications are identified by comparing relative retention times. Using these settings, a total of 187 potential modifications and common missense mutations were assessed on 31 fragments per replicate.

#### Total Proteomics

All MS/MS spectra were analyzed using Sequest (Thermo Fisher Scientific; version IseNode in Proteome Discoverer 3.0.1.27). Sequest was set up to search *Homo sapiens* (NcbiAV TaxID = 9606) (v2023–01–24; 186,728 total sequences including isoforms and known contaminants) assuming the digestion enzyme full trypsin. Sequest was searched with a fragment ion mass tolerance of 0.020 Da and a parent ion tolerance of 10.0 ppm. Carbamidomethyl of cysteine was specified in Sequest as a fixed modification. Met-loss of methionine, met-loss + Acetyl of methionine, oxidation of methionine, and acetyl of the n-terminus were specified in Sequest as variable modifications. Precursor ion intensity label-free quantitation was done using Proteome Discoverer. The two groups (infected and control) were compared using a “non-nested” study factor. Normalization was derived by using all peptides. Protein abundances were calculated by summed abundances, meaning the protein abundances are calculated by summing sample abundances of the connected peptide groups. One missed cleavage was allowed, the assumed target FDR was 0.01, and validation was based on a q value of 1.5 or greater.

#### Phosphoproteomics

Phosphoproteome data from Choudhary *et al*. (25) was retrieved from the Proteomics Identifications (PRIDE) Database under project PXD013171 along with the 6-plex tandem mass tag (TMT) marker weights and other parameters. In brief, peptides were injected into an Orbitrap fusion mass spectrometer (Thermo Electron) with a coupled Easy-nLC 1000 (Thermo Fisher Scientific), using Xcalibur (Thermo Fisher Scientific) for data-dependent acquisition (DDA). Selected precursor ions were excluded for 45 s and automated Synchronous Precursor Selection (SPS) was performed for MS3. Data was analyzed in Proteome Discoverer (version 2.2) (Thermo Fisher Scientific) and searched with Mascot (version 2.5.1) (Matrix Science) and Sequest against the UniProt human protein database (151,987 sequences, including potential contaminants and known isoforms). For the Orbitrap analyzer, fragment ion tolerance was set to 0.02 Da. Tolerance was 0.60 Da for the ion trap analyzer. Additionally, peptide mass tolerance was set to 10 ppm. Searches included static modifications of cysteine carbamidomethylation (+57.021 Da) and TMT tags on lysine residues and N-termini (+229.163 Da), as well as variable modifications of methionine oxidation (+15.995 Da) and N-terminal acetylation. Serine, threonine, and tyrosine phosphorylation were also assessed (+79.996 Da). Phosphorylation sites were identified with a ptmRS probability >90%. Phosphopeptides were assessed with the precursor neutral loss method. With reference to the expected mass based on precursor mass/charge ratio and charge state, neutral loss fragments were identified with a mass tolerance of 0.20 Da for the ion trap and 0.02 Da for the Orbitrap analyzer. The false discovery rate was restricted to <1% using a combination of the target strategy and linear discriminant analysis.

#### Protein Identification and Pathway Enrichment

Identified protein abundances were processed in R version 4.3.0 using RStudio. All packages used are either associated with Bioconductor (Bioconductor.org) or Tidyverse (26). Volcano plots are made using EnhancedVolcano (Bioconductor.org) and ggplot2 (27). Pathway enrichment was performed with clusterProfiler (28) for individual datasets. Metascape (29) was used to analyze patterns across the total proteomics and phosphoproteomics sets, as well as generate the heatmap and Circos plots. The cutoff for significantly enriched pathways was set at FDR less than or equal to 0.05.

#### Western Blotting

THP-1 cells were seeded at a density of 3.4 × 10^5^ cells/ml in T75 flasks, then differentiated and infected as described in the THP-1 Culture and Macrophage BCG Infection sections above. After incubation with BCG for 24 h at an MOI of 4, flasks were washed twice with warmed phosphate-buffered saline to remove excess bacteria. Cells were washed again with ice-cold Tris-buffered saline, placed on ice, and lysed with modified RIPA buffer (150 mM NaCl, 50 mM Tris-HCl, 0.1% Triton X-100, 0.5% sodium deoxycholate, 1 mM sodium fluoride, 0.1% SDS, 1 mM PMSF protease inhibitor, 1 tablet PhosSTOP phosphatase inhibitor per 20 ml buffer (Roche)). Flasks were scraped and the contents were sonicated three times at 50% amplitude for 2 s each (Fisherbrand Model 120 Sonic Dismembrator, Fisher Scientific). After centrifuging at 16,000*g* for 15 min at 4 °C, the supernatant was retained and a BCA assay was performed to calculate protein concentration. 15 ug of protein per sample was combined with 5X reducing loading buffer (50 mM Tris-HCl pH 6.8, 8% glycerol, 16% SDS, 4% beta-mercaptoethanol, 0.04% bromophenol blue), boiled at 95 °C for 5 min, then loaded onto a 12% SDS-PAGE gel.

After separation by electrophoresis, proteins were transferred to PVDF membranes (Immobilon-P, Millipore). Membranes were blocked in 3% BSA in TBST (20 mM Tris-HCl pH 7.5, 150 mM NaCl, 0.1% Tween-20) at room temperature for 1 h, then incubated overnight with primary antibodies at 4 °C in the blocking buffer. Antibodies against H4K8ac (#07–328) and HSP60 (#SAB4501464, 1:1000) were purchased from Sigma-Aldrich; H3K9me2 (#39239, 1:1000), H3K27ac (#39133), and MORF4L1 (#39361, 1:500) were purchased from Active Motif; TAF9 (#10544-1-AP, 1:1000) and SAMSN1 (#13063-1-AP, 1:500) were purchased from Proteintech; HSF1 (#PA3017, 1:1000) was purchased from Invitrogen; and beta-actin (#AHP1629, 1:2000) was purchased from Bio-Rad. HSP60 was used as the loading control for all antibodies except H3K9me2, for which beta-actin was used.

Membranes were washed five times with TBST, then incubated with a secondary antibody (Amersham ECL Rabbit IgG, HRP-linked whole Ab, 1:10,000 in PBS, Cytiva #NA934) for 1 h at room temperature. Finally, membranes were washed five times in TBST, then incubated for 1 min with Amersham ECL detection reagents (Cytiva) and imaged in an Amersham ImageQuant 800. Densitometry was performed in ImageJ.

## Results

### BCG Induces Expression of Immune and Epigenetic Proteins

**Fig. 1:**
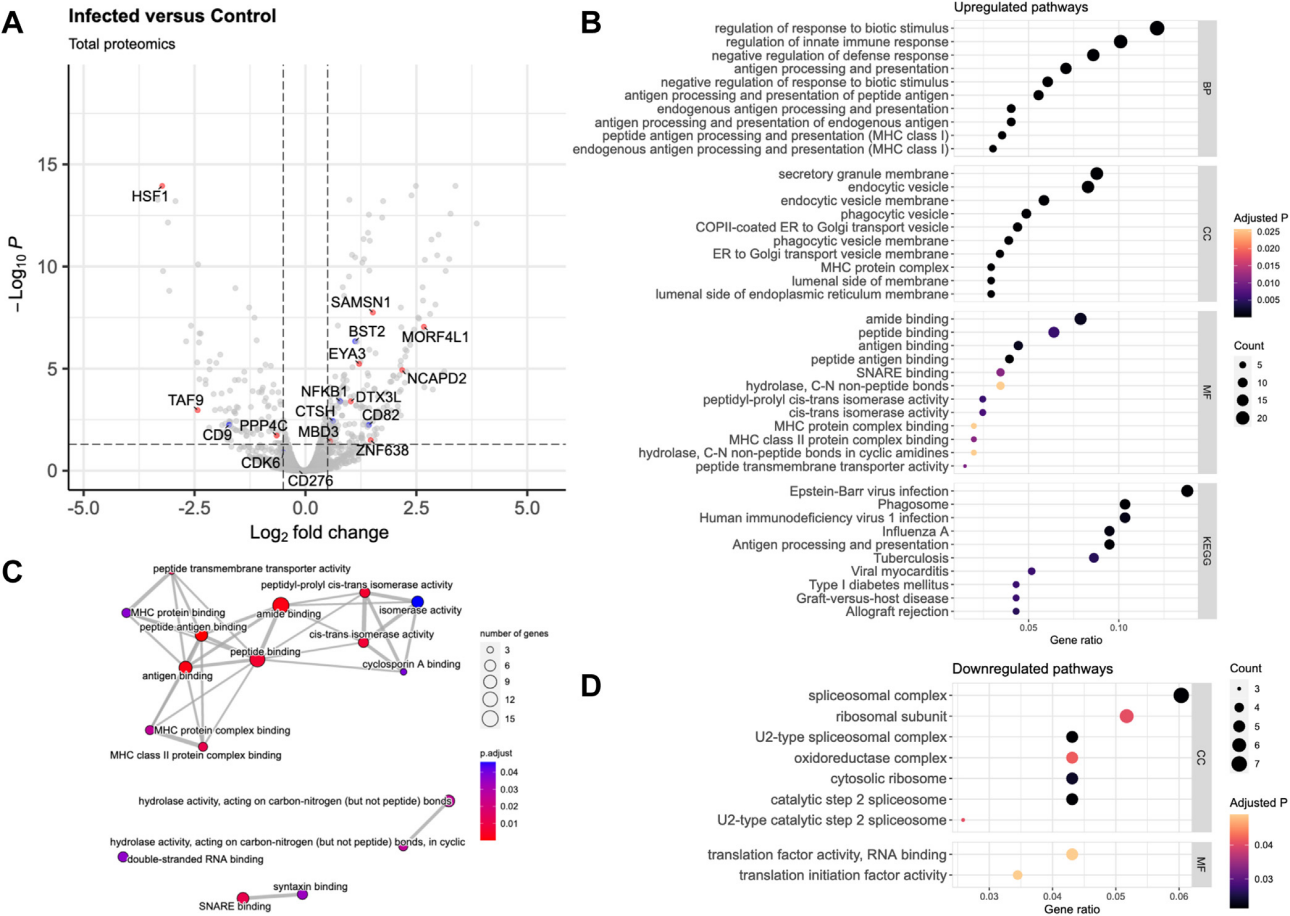
**Proteome of BCG-infected THP-1 macrophages shows differential expression of immune factors and histone modifiers**. *A*, a volcano plot of the total proteome highlighting chromatin-related proteins (*red*) and other proteins of interest in BCG-related applications (*blue*). *B*, upregulated GO and KEGG pathways with the 10 lowest *p* values in each category (GO-BP, GO-CC, GO-MF, KEGG). *C*, an interaction map of upregulated pathways in the molecular function subset. *D*, downregulated GO pathways. No downregulated enrichments were identified for GO-BP or KEGG.

A total of 3251 peptides were identified within the infected THP-1 total proteome (*p* < 0.05; fold-change >1.4). Figure 1*A* presents differential comparison of the proteomes of BCG-infected and uninfected THP-1 cells. As this study focuses on the beneficial and inflammatory effects of BCG, proteins of interest were separated into two major categories: chromatin-related (Fig. 1*A*, red) and other proteins heavily implied in other areas of BCG use (Fig. 1*A*, blue) such as cancer treatment or progression and HIV infection (Supplementary Files S1 and S2). Through this analysis, we identified a total of 17 unique peptides of particular interest. The chromatin-related category contains 10 peptides, primarily involved in DNA repair (DTX3L (30), EYA3 (31), HSF1 (32), MORF4L1 (33), PPP4C (34), TAF9 (35)) and histone acetylation or recruitment of histone condensation modifiers (MBD3 (36), MORF4L1 (37), NCAPD2 (38), TAF9 (39), ZNF638 (40)). We additionally list 7 peptides of interest in other areas: cancer-associated (BST2 (41), CD82 (42), CDK6 (43), CTSH (44), NFKB1 (45), SAMSN1 (46)) and HIV-related (BST2 (47), CD9 (48), CD82 (48), CD276 (49)).

To evaluate the differential regulation of pathways, we performed pathway enrichment using the clusterProfiler R package (Fig. 1, *B*–*D*). In these enrichment plots, the x-axis represents the ratio of coding genes found in the set to the total genes in each pathway. Figure 1*B* shows GO and KEGG enrichment, with GO pathways separated by subcategory. Multiple viral pathways are upregulated, most notably that of HIV-1 infection. We also see pathways typical of a bacteria-infected macrophage, such as antigen processing and presentation, granule production, and phagocytosis. The molecular function (MF) subcategory was expanded into an enrichment map (Fig. 1*C*) to further demonstrate the interconnected nature of these pathways. Figure 1*D* contains downregulated pathways, none of which were identified in the biological process GO category or in KEGG enrichment.

### Histone Proteomics Show Distinct Histone Tail Modifications in BCG-Infected Macrophages

The immunogenic effects of BCG in macrophages have been shown to be driven by epigenetic changes (50). To globally assess potential mechanisms, we examined histone tail PTMs after BCG infection.

**Fig. 2:**
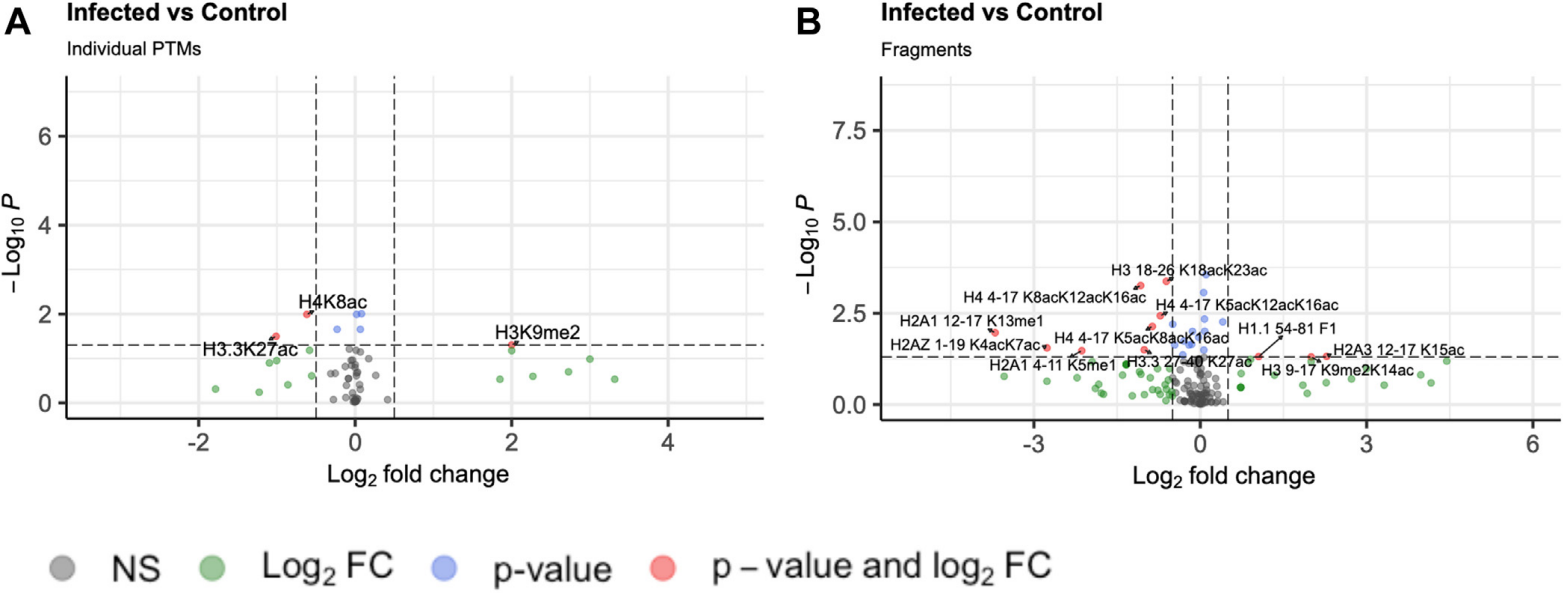
**Histone-focused proteomics showing a differential prevalence of histone PTMs.** Infected-control fold change cutoff 1.4, *p* value cutoff 0.05. *A*, a volcano plot showing overall differential expression and statistical significance of individual PTMs. *B*, co-occurrence of PTMs as found in digested fragments. F1 = GGVSLAALKKALAAAGYDVEKNNSR.

Figure 2*A* shows an analysis of the summed individual histone PTMs, regardless of the fragment on which they were identified. Figure 2*B* presents each fragment individually. The simultaneous presence of two PTMs can recruit a single epigenetic reader. As such, we include co-occurring PTMs to enable further discovery. Because simultaneous PTM presence can also be caused by two separate effectors, fragments were split and the individual PTMs contained were summed together. Using this method, H3 18 to 26 K18acK23ac would be classified both as H3K18ac and H3K23ac.

### Proteomics Findings are Validated in Immunoblotting

To confirm our findings of differential expression, we selected a subset of proteins from the total proteome and histone-focused analyses. Four proteins from the total proteome were chosen (TAF9, MORF4L1, HSF1, SAMSN1) and two from the histone-focused proteome were chosen (H3K9me2, H4K8ac) due to their proposed involvement in epigenetic modification and trained immunity. We also assessed the expression of H3K27ac, which is expected to be upregulated in trained immunity. The canonical form was selected due to the lack of antibodies that can identify H3.3K27ac.

**Fig. 3:**
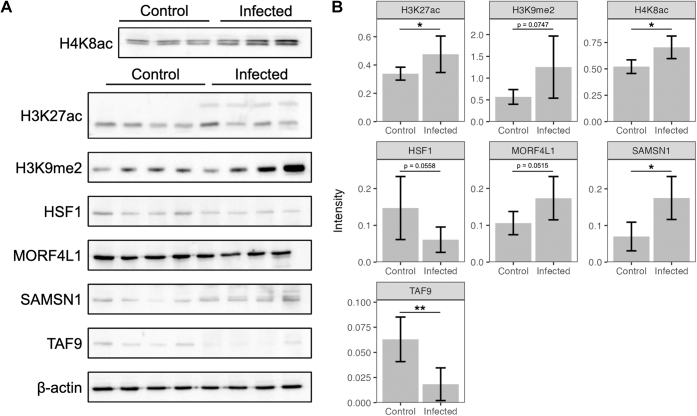
**Western blotting results**. *A*, images of target protein blots. *B*, plots of densitometry of target bands. ∗ indicates *p* < 0.05, ∗∗ indicates *p* < 0.01.

Immunoblotting supported similar differential expression in five of the seven proteins examined (Fig. 3). Due to the small sample size, we include here *p* values that did not reach the statistical significance threshold of 0.05 but were below 0.08 and may warrant consideration in this context. We positively identified MORF4L1 (*p* = 0.0515), TAF9 (*p* = 0.0087), SAMSN1 (*p* = 0.0260), H3K9me2 (*p* = 0.0747), and HSF1 (*p* = 0.0558) as supporting the differential expression patterns identified through proteomics approaches. H4K8ac (*p* = 0.0319) was upregulated through immunoblotting but downregulated through proteomics. Additionally, we assayed H3K27ac, which is traditionally upregulated in trained immunity. This was replicated in our findings (*p* = 0.0495).

### BCG Induces Differential Phosphorylation of Macrophage Immune and Epigenetic Pathways

Phosphorylation plays a significant role in activating and inactivating epigenetic readers and writers, as well as in propagating cellular signals in *Mycobacteria* infection. Choudhary *et al*. compared the phosphoproteomes of non-infected cells against those infected with BCG or virulent *Mycobacterium tuberculosis* H_37_Rv (25). A total of 6121 phosphopeptides were identified by Choudhary *et al*. in the BCG-control dataset. Of these peptides, 1325 were both statistically significant (*p* < 0.05) and had a fold change greater than 1.4. Secondary bioinformatic analyses of BCG and control phosphoproteome revealed pathways of interest for epigenetic regulation.

**Fig. 4:**
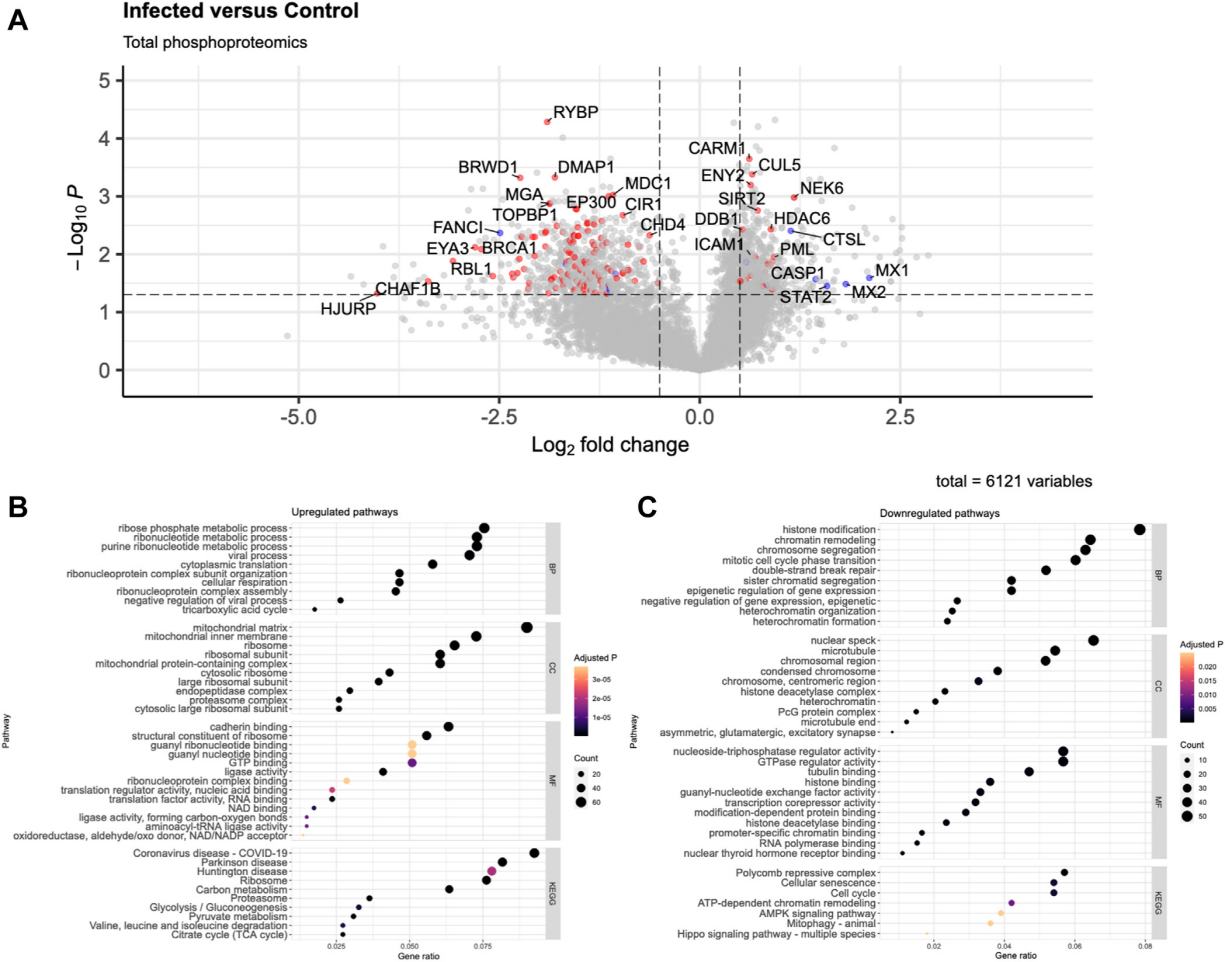
**A plot detailing phosphoprotein enrichment**. *A*, a volcano plot with the same categories as in Figure 1 (chromatin-related in *red*; other proteins of interest in *blue*). *B*, upregulated pathways identified through GO and KEGG enrichment. *C*, downregulated pathways are identified in the same way.

In Figure 4*A*, we present the phosphoproteome in a volcano plot. Highlighted in red are 135 proteins involved in chromatin regulation and modification. Blue points indicate 14 additional proteins of interest involved in infection response and cancer growth.

Upregulated GO and KEGG pathways shown in Figure 4*B* include glycolysis, TCA cycle, and metabolism. We also find some unusual pathways, including neurodegenerative diseases and viral pathogenesis. The downregulated pathways as shown in Figure 4*C* lean heavily towards epigenetic regulation, including chromatin remodeling and activity of Polycomb repressive complexes.

### Comparative Pathway Enrichment Analysis Reveals Functional Similarities Between Data Sets

A major difference separating the proteome and phosphoproteome datasets is the time between infection and peptide harvest. Timelines for trained immunity are variable, and common intervals range from 24 h to several days (50, 51). To compare the responses obtained for variable timelines, we investigate pathway enrichment and functional similarities across both proteome and phosphoproteome datasets.

**Fig. 5:**
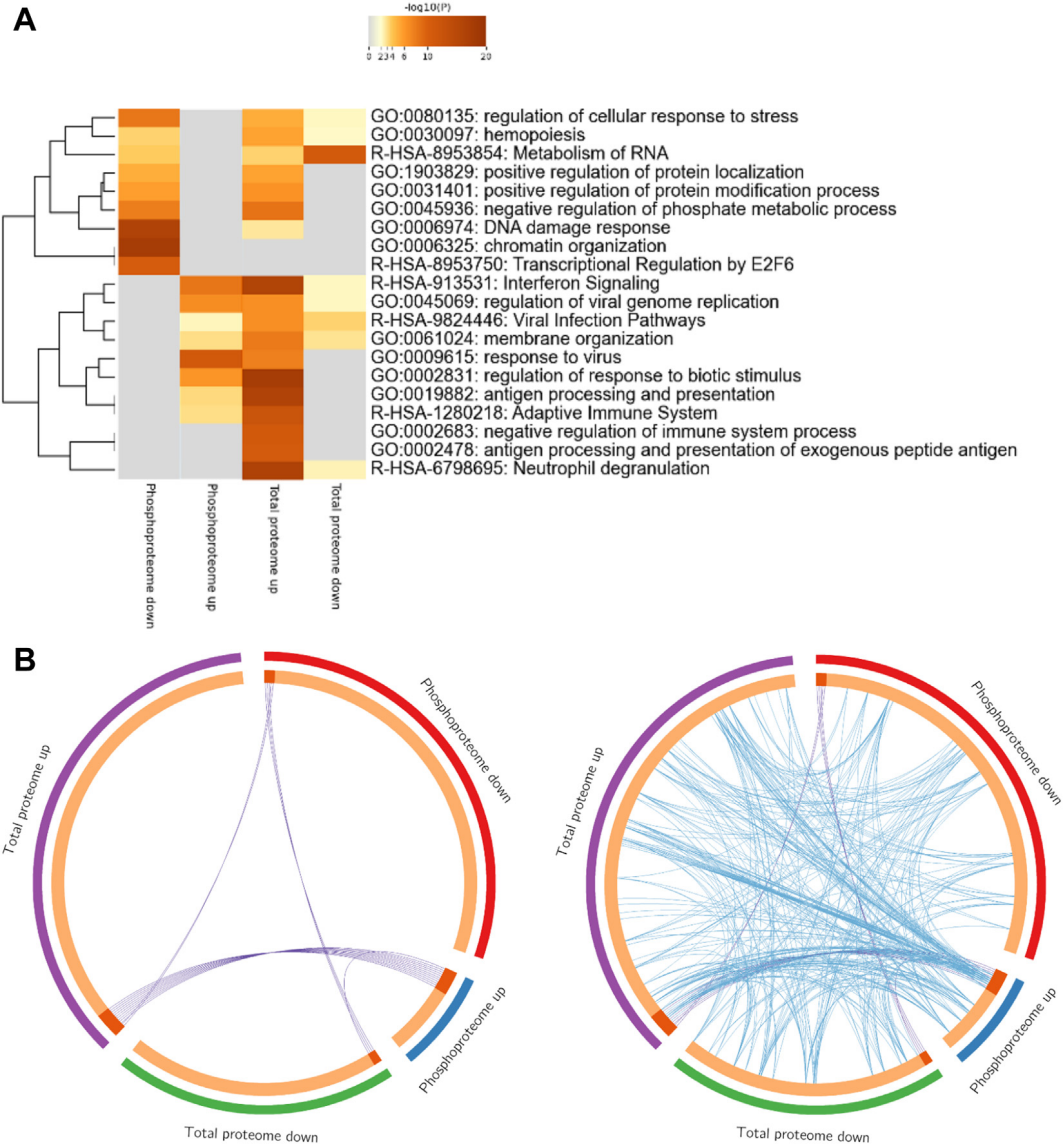
**Metascape gene annotation and analysis**. *A*, a dendrogram and heatmap of enriched gene terms in each category. *B*, Circos plots showing overlapping genes from each category. *Blue* indicates functional overlap, while purple indicates genes directly shared between categories.

Figure 5*A* shows a dendrogram and heatmap comparing enriched terms in both the proteome and phosphoproteome. These include GO terms, Reactome pathways, Gene Set Enrichment Analysis (GSEA) terms, and WikiPathways networks. Overall, the total proteome set has more upregulated pathways involving immediate response to infection, while the phosphoproteome set has more downregulated pathways involving epigenetics and chromatin organization.

Given that one gene can be involved in numerous cellular functions and pathological processes, we use a Circos plot (Fig. 5*B*) to visualize the overlap between shared genes or functions.

Purple lines connect proteins that are included in multiple sets, while blue lines indicate shared gene ontology function. This also serves to highlight that while the direct overlap of proteins may be small, implied functional overlap is much larger. Despite differences in stimulation parameters in BCG exposure between the proteome and phosphoproteome, we identify numerous common functional changes.

### Multilevel Analysis Reveals an Epigenetic Interaction Network

From data obtained in the analysis of the histone-focused proteome, total proteome, and phosphoproteome, we highlight key players that may contribute to BCG-mediated epigenetic modifications. Through systematic examination and identification of epigenetic writers and erasers, their subunits, and substrates (histone post-translational modifications) (Table 1) we can determine new pathways for potential BCG-induced activation. We identify both histone acetyltransferases (HATs) and histone deacetylases (HDACs), each of which is linked to epigenetic activity. In the phosphoproteome dataset, we also identify members of the sirtuin family, lysine deacetylases with diverse cellular targets (52).

**Table 1:** Epigenetic networks identified across the histone-focused proteome, total proteome, and phosphoproteome datasets

Modifier/complex identified	Differentially identified components	Differentially identified substrate PTMs
NuA4 (HAT) (72)	*DMAP1, EP300, EP400, KANSL1, KANSL3,***MORF4L1**, *MRGBP*	**H2AK15ac**, **H3K14ac**, *H4K5ac, H4K8ac, H4K12ac, H4K16ac*
NuRD (HDAC) (52)	*CHD4, GATAD2B, MTA1,***MBD2, MBD3**	H2B nonspecific, H3 nonspecific
NSL (HAT) (73)	*KANSL1, KANSL3, PHF20L1*	*H4K5ac, H4K8ac*
Sin3A (HDAC) (52)	**MORF4L1**, *SAP130, REST*	H4 nonspecific
SIRT2 (HDAC) (53, 56)	*SIRT2*	Potential: H3 nonspecific, H4 nonspecific
SIRT6 (HDAC) (69, 74)	*SIRT6*	Potential: H3 nonspecific, H4 nonspecific

Bolded text indicates an increase in infected cells; italicized text indicates a decrease.

In addition to the known substrates, we identify multiple epigenetic writers and erasers with significantly enriched presence whose representative substrates were not affected after BCG infection (Table 2). Within this category, lysine methyltransferases (KMTs), as well as Polycomb group (PcG) members, are of interest as their function ranges from ubiquitin ligation to lysine trimethylation.

**Table 2:** Epigenetic effectors identified without substrates

Modifier/complex identified	Differentially identified components	Expected substrate PTMs
MLL1 (KMT)	*ASH2L, CHD8, KANSL1,***MEN1**, *MGA*, **RUVBL1**, *TAF9*	H3K4me (75)
MLL3/4 (KMT)	*ASH2L, KMT2C, KMT2D, NCOA6*	H3K4me (75)
Other PcG members	*ASXL1, ASXL2, BAP1, EZH2, PHC2, RING1, SCML2*	Various
PR-DUB (deubiquitinase)	*ASXL1, ASXL2, BAP1, FOXK1, FOXK2*	H2AK119ub1 (76)
PRC1 (ubiquitin ligase)	*BMI1, PHC2, RING1*	H2AK119ub1 (36)

Bolded text indicates an increase in infected cells; italicized text indicates a decrease.

**Fig. 6:**
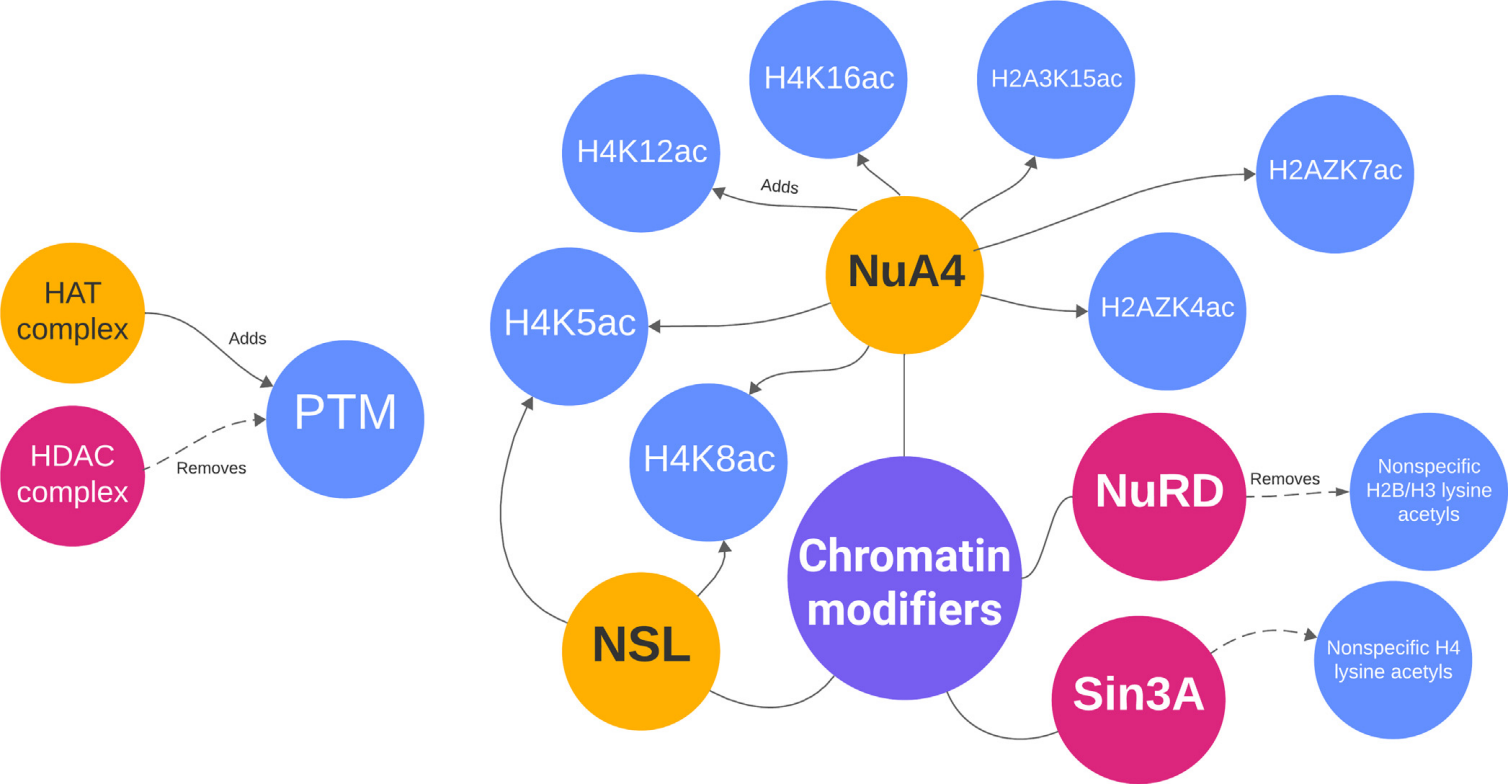
**Interaction network of differentially present epigenetic effectors and their PTMs.***Yellow circles* represent HATs, *pink circles* represent HDACs, and blue circles represent PTMs. HAT: histone acetyltransferase; HDAC: histone deacetylase; NSL: non-specific lethal complex; NuRD: nucleosome remodeling and deacetylase complex; PTM: post-translational modification.

In Figure 6, we present a novel network of selected modifiers and their target PTMs in BCG-infected macrophages. Derived from Tables 1 and 2, this network visualizes how chromatin modifiers connect to specific histone targets. SIRT2 can be identified both in the nucleus and cytoplasm, where it acts on targets ranging from tubulin to histones. It has been closely linked to the prognosis of cancers including gliomas (53), chemoresistance (54), regulation of cellular metabolism, inflammation, and oxidative stress (55). Furthermore, it is proposed to directly influence tuberculosis infection both by altering transcription through epigenetic activities and through influencing NFκB signaling (56). SIRT6 mainly functions in the nucleus, where it can deacetylate residues on H3 (57). However, it also functions as an ADP-ribosyl-transferase, key in the activation of PARP1 in the repair of double-strand breaks in genomic DNA (58). SIRT6 influences glycolysis and glucose metabolism and is a *Myc* co-repressor, indicating a potential role in the metabolic activity of cancer cells (58, 59). Both SIRT2 and SIRT6 have documented involvement in other aspects of epigenetic modification and trained immunity, so we exclude both from the network to avoid the influence of factors beyond the direct addition and removal of histone PTMs.

## Discussion

BCG inoculation has been shown to cause lasting immunological effects, with wide-ranging implications. As BCG has been shown to elicit its immunomodulatory effects *via* regulation at the histone level, it is important to characterize histone post-translational modifications in affected immune cells. This has previously been interrogated in a targeted manner, assessing histone PTMs through analysis of a few well-established marks (60). To optimally leverage the benefits of BCG, the immunological processes induced in macrophages upon exposure must be identified and mechanisms elucidated. Here we employ mass-spectrometry-based proteomics with an *in vitro* infection model to globally profile peptides in the total, phospho, and histone proteome. Through individual analyses of multi-level data, we highlight differentially expressed proteins of interest in BCG infection and identify several pathways that suggest novel mechanisms driving BCG-mediated immunogenicity. Ultimately, we unveil pathways, epigenetic machinery, and histone targets in BCG stimulated immune cells which are necessary to understand mechanisms of immunomodulation.

Within the proteome data, we identify trends confirming the involvement of anti-viral pathways in the cellular response to BCG infection. We also identify pathways typical in bacterial infection, such as antigen processing and presentation. These findings in the total proteome set align with many previous findings in mycobacterial infection, including those of Choudhary *et al*. (61, 62). Notably, there are relatively few downregulated GO or KEGG pathways in this sample. We suggest that this may be a result of the highly activated state a macrophage is in shortly after infection. Additionally, MHC class II binding is upregulated, which implies the involvement of CD4-positive T cells that would then stimulate an antibody response against bacterial infection. This may be involved in BCG-HIV interactions, as CD4-positive T cells are a primary target for HIV infection.

For histone proteomics, we identify several histones that are modified within 24 h of BCG stimulation. Individual PTMs that were statistically significant include H3.3K27ac, H4K8ac, and H3K9me2. H4K8ac can be edited by CBP/KAT3A, p300/KAT3B, NSL, and NuA4 acetyltransferases. This is supported by Tables 1 and 2, which list components of NSL and NuA4 that were identified in the total proteome and phosphoproteome data.

Of the 3 epigenetic marks that are firmly associated with trained immunity in primary macrophages (14, 50), we identify only H3K27ac. However, within this experiment, we highlight initial changes to the epigenome, without restimulation. It is also important to note that different training stimuli generate unique training programs (51), and specific epigenetic signatures of each stimulus have not been clearly defined. Furthermore, this data implies that some histone marks may be primed from initial stimulation, before restimulation with other pathogens.

For our targeted validation, immunoblotting supported the findings from mass spectrometry, apart from H4K8ac. H4K8ac was expected to be downregulated in infected cells from mass spectrometry data but was found to be upregulated by immunoblotting. Immunoblotting for H3K27ac was increased in infected macrophages, showing that this traditional marker for trained immunity is primed upon initial infection. Within our proteomics data, we found the variant H3.3K27ac less abundant, however, available antibodies do not discriminate between the two. Additionally, histone fragments are light (H3K27ac appears at about 15 kDa on Western blots; H4K8ac appears at 8 kDa), which can present a challenge for Western Blotting techniques. We highlight this as an advantage of mass spectrometry-based approaches over targeted blots.

In the phosphoproteome data, we identify pathways such as response to antigen exposure, and interferon signaling. We additionally see the involvement of epigenetic pathways and effectors such as Polycomb complex activity, chromatin remodeling, and heterochromatin formation. Through these data, we can determine how epigenetic modifiers are also regulated via phosphorylation after BCG exposure. Of note, in the phosphoproteome set, we see that pathways associated with HIV-1 infection and immune conditions including graft-versus-host disease and type 1 diabetes mellitus are enriched.

In comparing total proteome and phosphoproteome pathway enrichment, we found that the total proteome sample is mainly enriched in pathways directly involved in response to infection, while the phosphoproteome sample has significantly more processes that involve chromatin organization and epigenetic regulation.

Figure 6 presents a novel network of epigenetic editors and their substrates that are supported by this data. We highlight acetyltransferases and deacetylases, which have been very closely linked to establishing the training response, and show a unique high-level view of the multiple histone PTMs they change to create the resulting phenotype. In addition to the network defined in Figure 6, we also identified multiple effectors that were significantly enriched but did not have corresponding substrates (Table 2). These will require further investigation to confirm individual complex activity. Furthermore, future work should explore direct links between these complexes and marks with associated genes through targeted analyses such as ChIP-PCR to additionally elucidate aspects of the trained immunity network.

Notably, we also identify within this network and the associated data several epigenetic actors associated with HIV infection, latency, reactivation, persistence, and comorbidities (63). This group includes EZH2 (59, 64), both PRC1 and PRC2 (65), SIRT2 and SIRT6 (66), modifications to histones including H3K9 (67), and histone-rearranging complexes (68). These molecules should drive additional investigations as potential effectors to reduce HIV latency and persistence as an obstacle to a cure.

The exclusion of SIRT2 and SIRT6 from this network was primarily driven by their widespread influence on cellular metabolism and other aspects of epigenetic modification (53, 57, 69, 70). The only sirtuin examined in the context of trained immunity thus far is SIRT1 (71), and the epigenetic influences of neither SIRT2 nor SIRT6 have been investigated in sufficient depth given their extensive reach within the cell. Given that we have identified differential expression in this context, further research should be done to identify the role of each molecule in the mechanisms of trained immunity.

As indicated by Choudhary *et al*. and additional literature (51), training takes several hours to establish, and the effects of the resting period begin to plateau past several days. This phenomenon is likely reflected in our proteomics data when compared to the phosphoproteomics data. As mentioned briefly, macrophages are in a highly activated state after infection with the primary focus being to clear pathogens. This is supported by the multiple anti-pathogen pathways upregulated in the proteomics data, sampled at 24 h after infection. However, as shown by the phosphoproteomics data, epigenetic modification pathways are highly downregulated 72 h after infection. This likely indicates the onset of the plateau period, where cells have already made the epigenetic modifications required to shift to the trained phenotype.

Differences between THP-1 cells (derived from acute myeloid leukemia) and primary macrophages are also important to consider within the context of this work. THP-1 cells have been shown to have mutations in genetic regions encoding key epigenetic modifiers, including MLL1 (KMT2A), an essential writer of H3K4 methylation. This mark was not found to be differentially enriched in our data, potentially due to a lack of a functional epigenetic writer.

These findings identify multiple directions for future work. First, the effectors identified can be mapped to specific genes, strengthening our understanding of the induction of trained immunity. Additionally, within the context of TB and HIV, which are often co-occurring, these findings provide data to identify improved therapeutic targets by highlighting unique peptides and pathways enriched in various disease processes after BCG exposure. Our research elucidates specific effectors with the potential to drive the development of trained immunity phenotypes. Through this multilevel proteomic analysis, we identify plausible mechanisms of BCG-mediated trained immunity. Furthermore, we identify several key proteins and pathways important to examine in BCG-specific applications.

## Data Availability

Phosphoproteome data is available *via* ProteomeXchange with identifier PXD013171.

The epiproteomics and proteomics mass spectrometry data have been deposited to the ProteomeXchange Consortium *via* the PRIDE (23) partner repository with the dataset identifier PXD051187 and 10.6019/PXD051187.

## Supplemental data

This article contains supplemental data.

## Conflict of interest

The authors declare that they have no conflicts of interest with the contents of this article.

## Supplementary Data

File S1 Total proteome data as output from Proteome Discoverer.

File S2 Proteins of interest in the total proteome (*p*-value <0.05, fold change >1.4).

File S3 Proteins of interest in the phosphoproteome (*p*-value <0.05, fold change >1.4).
